# Families’ situation of caring for a child with a chronic condition: a mixed methods study*

**DOI:** 10.1590/1980-220X-REEUSP-2023-0304en

**Published:** 2024-03-08

**Authors:** Melissa Joice de Abreu Felizardo, Maísa Mara Lopes Macêdo, Nayara Luiza Henriques, Sérgio Deodato, Elysângela Dittz Duarte

**Affiliations:** 1 Universidade Federal de Minas Gerais, Escola de Enfermagem, Departamento de Enfermagem Materno-Infantil e Saúde Pública, Belo Horizonte, MG, Brazil.; 2 Universidade Católica Portuguesa, Faculdade de Ciência da Saúde e Enfermagem. Centro de Investigação Interdisciplinar em Saúde. Lisboa, Portugal.

**Keywords:** Family Nursing, Pediatric Nursing, Family, Child Care, Chronic Disease, Enfermería de la Familia, Enfermería Pediátrica, Familia, Cuidado del Niño, Enfermedad Crónica, Enfermagem Familiar, Enfermagem Pediátrica, Família, Cuidado da Criança, Doença Crônica

## Abstract

**Objective:**

To analyze the meanings attributed by family members to the situation of caring for a child with a chronic condition (CCC), in the light of the Family Management Style Framework (FMSF).

**Method:**

A mixed-methods, parallel-convergent study, guided by the FMSF theoretical framework, using the conceptual component “Definition of the Situation”. Fifty-three CCC families took part. Data was collected using a semi-structured interview, a questionnaire to characterize the participants and a Family Management Measure scale. Descriptive and inferential statistical analysis was carried out on the quantitative data and the qualitative data was subjected to deductive thematic analysis.

**Results:**

Family members reported a view of normality in relation to CCC, also verified by the Child’s Daily Life scale. However, they indicate the repercussions of the chronic condition on the family, and that they devote more attention and time to meeting the child’s care needs, which was also verified in the View of the Impact of the Condition and Management Effort scales.

**Conclusion:**

Families have a positive view of the situation of caring for CCC at home, but point out some negative effects, such as the greater time spent caring for the child.

## RESUMO

**Objetivo:**

Analisar os significados atribuídos por familiares a situação de cuidar de uma criança com condição crônica (CCC), à luz do Family Management Style Framework (FMSF).

**Método:**

Pesquisa de métodos mistos, do tipo paralelo convergente, orientada pelo referencial teórico FMSF, utilizando o componente conceitual “Definição da Situação”. Participaram 53 famílias de CCC. Os dados foram coletados por entrevista semiestruturada, questionário de caracterização dos participantes e escala de Medida de Manejo Familiar. Realizou-se análise estatística descritiva e inferencial dos dados quantitativos e os dados qualitativos foram submetidos à análise temática do tipo dedutiva.

**Resultados:**

Familiares referem uma visão de normalidade em relação à CCC, verificada também pela escala Vida Diária da Criança. Contudo, indicam as repercussões da condição crônica na família, e que dedicam maior atenção e tempo para atender às necessidades de cuidado da criança, também verificado nas escalas de Visão do Impacto da Condição e Esforço de Manejo.

**Conclusão:**

As famílias possuem uma visão positiva da situação de cuidar das CCC no domicílio, apontando, contudo, alguns efeitos negativos, como é o caso do maior tempo despendido na atenção à criança.

## RESUMEN

**Objetivo:**

Analizar los significados atribuidos por los familiares a la situación de cuidado de un hijo con una condición crónica (CCC), a la luz del Marco de Estilos de Gestión Familiar (FMSF).

**Método:**

Estudio de métodos mixtos, paralelo-convergente, orientado por el marco teórico del FMSF, utilizando el componente conceptual “Definición de la Situación”. Participaron 53 familias CCC. Los datos se recogieron mediante una entrevista semiestructurada, un cuestionario para caracterizar a los participantes y una escala de Medida de Gestión Familiar. Se analizaron estadísticas descriptivas e inferenciales para los datos cuantitativos y los datos cualitativos se sometieron a un análisis temático deductivo..

**Resultados:**

Los familiares relataron una visión de normalidad en relación al CCC, verificada también por la escala de Vida Cotidiana del Niño. Sin embargo, señalan las repercusiones de la condición crónica en la familia, y que dedican más atención y tiempo a la atención de las necesidades de cuidado del niño, lo que también fue verificado en las escalas Visión del Impacto de la Condición y Esfuerzo de Gestión.

**Conclusión:**

Las familias tienen una visión positiva de la situación de cuidar de CCC en casa, pero señalan algunos efectos negativos, como el mayor tiempo dedicado al cuidado del niño.

## INTRODUCTION

The onset of a chronic condition in a child is a complex situation, capable of activating feelings of fear, guilt, incapacity and doubts about care in families^(1,2)^. After experiencing the moment of diagnosis, the family needs to come to terms with the situation and create strategies aimed at developing the child’s care and treatment^(1,2)^. Although all family members can suffer the repercussions of the presence of a chronic condition, mothers are the ones who mostly dedicate themselves fully to care, assuming the role of main caregiver^(3)^.

Although the care of a child with a chronic condition (CCC) is mainly carried out by the main caregiver, efforts are required from other family members to maintain a balance in the child’s health condition, seeking to bring it closer to that expected of other children of the same age^(4)^. The coordination of care creates a stable and peaceful family environment, enabling those involved to live their lives in the best possible way, allowing them to have a perception of normality even with the daily demands of caring for their children^(5)^.

It is recognized that the family as a whole and its individual members need to adapt to the context of the child’s chronicity. This adaptation is influenced by aspects related to the complexity and severity of the child’s health condition, the social support received during the course of care^(6)^ and access to resources, such as support from health system professionals^(7)^. In addition, the family’s assessment of the child’s care situation and their ability to manage it can also change their adaptation to the care situation over time and consequently family functioning^(8,9)^.

The family’s assessment of the care needs of the CCC and how they respond to these needs is called Family Management^(8)^. Family management is addressed in the Family Management Style Framework (FMSF)^(8)^, translated and validated for Brazil as “*Modelo de Estilo de Manejo Familiar*”^(9)^. The main components of this framework are Situation Definition, Management Behavior and Perceived Consequences. Specifically, the Definition of the Situation, which is the subject of this study, refers to how the family perceives the child and their health condition and how their perception can interfere in the management of childcare^(8)^.

The *Situation Definition* component addresses the following aspects: i) *Identity of the Child* - the family’s view of the child and how much the focus is on the chronic condition or normality, on abilities or vulnerabilities^(8)^; ii) *View of the illness* - the family’s beliefs about the cause, severity, prognosis and course of the chronic condition^(8)^; iii) *Management Mentality* - the family’s view of the ease or difficulty of complying with the treatment regimen and their ability to manage it effectively^(8)^; iv) *Mutuality between parents* - the beliefs of the caregivers who occupy the role of the child’s father and mother about the extent to which they have shared or divergent perspectives on the child and the management of the disease^(8)^.

This study looks at the *Situation Definition* component, i.e. what it means for the family to care for a child with a chronic condition. Even though this research considers the family as a system encompassing different members who live in the same place^(8)^, the survey was carried out with the respondents as the main caregivers (people who take on most of the child’s care)^(3)^. This is because it is these caregivers who have information that makes it possible to explore the aspect of interest in the study, which is the relationship between one of its members (the child) and the family as a system.

It is based on the assumption that the meaning attributed by the family to the child and his or her health condition can change family management. Families that focus on the child’s chronic condition and weaknesses may experience greater difficulties in providing care, with possible compromises in meeting the needs of children and other family members. Therefore, knowing what families experience when caring for CCC at home can help identify aspects that are common to families and that deserve to be observed in the context of care by professionals in order to facilitate this process.

Based on the above, the research question was: What is the meaning attributed by the family to the situation of caring for a child with a chronic condition, considering the child with the chronic condition, their health condition and the management carried out by the family? The aim was to analyze the meanings attributed by family members to the situation of caring for a child with a chronic condition, in the light of the theoretical framework Family Management Style Framework (FMSF).

## METHOD

### Study Design

This is a study using convergent parallel mixed methods^(10)^ using the theoretical framework of the Family Management Style Model^(8)^. In this investigation, the quantitative study was cross-sectional and descriptive, while the qualitative approach was descriptive^(11)^. Both approaches were given equal weight.

### Study Site

The participating families were identified through the hospitalization records of children discharged from the Neonatal Intensive Care Unit (NICU) of two reference hospitals for maternal and child health care in the city of Belo Horizonte, in the state of Minas Gerais, Brazil. Participants included families living in Belo Horizonte and its metropolitan region, whose data was collected at home, as well as families living in different cities in the state of Minas Gerais, whose data was collected by telephone.

### Population

Fifty-three families of CCCs who had left the Neonatal Intensive Care Unit were interviewed. It should be noted that the representatives of the family system who took part in the interviews were the mother, father and aunt and grandmother.

### Selection Criteria

The criteria for including family members in the study were: being responsible for the child’s care, living in the same household as the child, being over 18 years old. The criterion for excluding participants was having psychological or cognitive alterations that would compromise data collection.

### Sample Definition

The families were identified from the hospital admission records, considering the children who were discharged from the Neonatal Intensive Care Unit (NICU) between December 2016 and December 2017 from two reference hospitals for maternal and child health care, totaling 263 children at the Federal hospital and 852 children at the Philanthropic Hospital. The period was defined to enable the identification of children aged between 2 years and 2 years 11 months and 29 days. The age group was previously defined on the basis that it is at this age that alterations in neuropsychomotor development are most commonly identified^(12)^. Subsequently, each family member was contacted by telephone between October 2019 and May 2020 and the Questionnaire for the Identification of CCC - Revised (QuICCC-R)^(13)^ was applied to confirm chronicity in childhood. It should be noted that 286 family members were successfully contacted, of whom 218 did not have a chronic condition according to the QuICCC-R, 5 children had died and 8 families refused to take part in the study, one of whom had twin children. As a result, the study sample consisted of 53 families, one of which had twins, totaling 54 children.

### Data Collection

Data was collected between October 2019 and May 2020 through interviews with the families. These interviews were scheduled in advance, on a day and time of the family’s preference, after the first telephone contact was made to identify the chronic condition. Families living in Belo Horizonte and the metropolitan region were interviewed at home, while families living in other cities in Minas Gerais were interviewed by telephone.

Quantitative data was obtained by applying a questionnaire to characterize the sociodemographic features of the families and by employing the Family Management Measure scale, a version adapted and validated for Brazil^(8,9)^. This instrument contains 53 items in two sections. Section 1 consists of five scales: Child’s daily life, Management ability, Management effort, Family difficulty and View of the impact of the condition. Section 2 is made up of the Mutuality between parents’ dimension, which will be answered when there are adult partners in the same household. It should be noted that this analysis used the scales Child’s Daily Life (CDL), View of the Impact of the Condition (VIC) and Management Effort (ME), scales corresponding to each of the aspects of the Situation Definition component^(8,9,14)^. The Mutuality between Parents’ scale, although part of the Definition of the Situation dimension, was not used in this analysis, without detriment to the study’s objective, as it refers to the caregivers’ beliefs and the shared perspective for caring for the child, when both are participants.

The answers to the scales used were obtained using a Likert scale ranging from totally disagree (1) to totally agree (5). The CDL scale contains five questions that measure the parents’ perception of the child and their daily lives. Higher scores indicate a normal life for the child despite the condition. Its minimum and maximum scores are 5 and 25 respectively. The VIC scale contains 10 items and measures the severity of the chronic condition in the family environment and the repercussions for the child and family members. Higher scores indicate a high level of concern about the condition. The minimum score is 10 and the maximum is 50. The ME scale has 4 questions and addresses the actions needed to manage the condition. Higher scores indicate more time and effort in managing the chronic condition. The minimum score is 4 and the maximum is 20^(14)^.

The version of the Family Management Measure scale validated for Brazil showed a Cronbach’s alpha (0.89), with a value of 0.78 for the Child’s Daily Life scale, 0.56 for the View of the Impact of the Condition and 0.51 for the Management Effort scale, achieving an acceptable fit for validation standards^(9)^.

Qualitative data was collected using a semi-structured interview based on the FSMF framework^(8)^. The aim was to develop questions that would provide information on each of the components of the framework used in this study: i) Identity of the child: Tell me about your family’s day-to-day life, What do you think the day-to-day care of (Child’s Name) is like compared to other children of the same age? why? ii) Management mentality: In the family context, were there any difficulties or facilities in caring for the child at home? If so, which ones? What is the approximate time required to care for (child’s name)?; 3) View of the illness: What is it like for you to deal with (child’s name)’s condition? In the future, what do you expect to be the demand for care for (child’s name)’s chronic condition?

### Data Analysis and Processing

The 53 interviews were conducted by the first author of the study, lasting an average of 52 minutes. The quantitative data was stored in a database on the Survio^®^ Platform. The data was double-entered for accuracy. The final version of this database was exported to Stata^®^ software version 15.0. The sociodemographic variables were subjected to calculation of means and dispersion measures (standard deviation, minimum and maximum) and calculation of absolute and percentage distributions. The scores of the scales, Child’s Daily Life, View of the Impact of the Condition and Management Effort of the Family Management Measure instrument were calculated according to the guidelines recommended in the literature^(14)^.

The qualitative data obtained through the semi-structured interview was audio-recorded and transcribed in full by the first author of the study. The text was revised by comparing it with the audio. MaxQDA^®^ software, version 20.0, was used for anonymized data storage and analysis. Quantitative variables were also exported to this software for subsequent cross-referencing of quantitative and qualitative data.

A Deductive Thematic Analysis was adopted for this study^(15)^, defining one code (Definition of the Situation) and three sub-codes (Identity of the Child; View of the Illness; Management Mentality) based on the dimensions and concepts derived from the theoretical framework of the Family Management Style Model^(8)^. These codes were applied simultaneously and independently by two researchers. Subsequently, the coding was reviewed and validated by a third researcher, who also established consensus in cases of divergence in the application of the codes. To check that the coding was consistent, a coded interview was sent to an external researcher with experience in using the theoretical framework. Finally, the codifications were compared and the rate of agreement between the researchers was verified, with a Kappa coefficient of 0.92^(16)^.

### Ethical Aspects

The research was submitted and cleared in 2019 by the Research Ethics Committee of the institutions where the study was carried out, according to opinion 3.508.414. In 2020, an amendment was submitted to the Research Ethics Committee to allow data to be collected via telephone contact. The amendment was approved in 2020, according to opinion 4.103.704. In order to access the hospitalization records, the researchers signed the Data Use Consent Form (DUCF). The study participants had their consent to participate recorded and stored on secure, anonymized media after reading the Informed Consent Form (ICF). As this is an investigation involving human beings, this study complies with the aspects contained in Resolutions 466/12 and 580/2018 of the National Health Council^(17,18)^.To ensure the anonymity and confidentiality of the participants, the interviews were anonymized by replacing the names with alphanumeric codes, composed of the letters M, P, T and C which refer to mother, father, aunt and child, respectively. These codes were followed by a number indicating the order of inclusion in the study (e.g. M2).

## RESULTS

The study encompassed fifty-three families of CCC who had left the NICU. Of that total, 88.68% (n = 47) of the participants were the children’s mothers, 7.55% (n = 4) were fathers and 3.77% (n = 2) were great-aunts. The average age of the family members was 33.58 years. With regard to place of residence, 69.81% (n = 37) lived in the interior of the state of Minas Gerais. With regard to schooling, the majority of participants, 47.17% (n = 25), had completed high school/incomplete higher education. With regard to profession, 58.49% (n = 31) declared themselves to be in the technical/manual category; 9.43% (n = 9) in the administrative category; 30.190% (n = 16) in the household category; 1.89% (n = 1) did not state their professional category. With regard to marital status, 77.36% (n = 41) lived with their partner. The average number of people living in the same household was 3.90. The average family income was 2,076.83 reais, equivalent to 1.98 minimum wages at the time of data collection. Of the 53 families, 34 children were discharged from the Philanthropic Hospital (64.15%) and 19 from the Federal Hospital (35.85%), with varying CCCs and different repercussions on their health status and levels of dependency. Most of the children were male (66.04%), with an average age (in years) of 2.73 years.

Most of the participants (71.70%, n = 38) scored above 15 on the CDL scale, indicating a view of normality in relation to their children, despite their chronic condition. 24.53% (13) participants scored below 15, and two (3.77%) scored 15 on the same scale.

Based on the interview data, it can be seen that of all the participants with the highest scores on the CDL scale, with the exception of 38 participants, all the others mentioned some aspect related to the child’s limitations or vulnerability (Chart 1). These included those determined by their chronic condition and characterized by the need to use technological devices, medication and limited mobility.

**Chart 1:** Quantitative results obtained using the Child’s Daily Life Scale and qualitative data from the Child’s Identity category – Belo Horizonte, MG, Brazil, 2020.

Child’s Dailife Score (minimum: 5 maximum: 25; mean: 15)^a^ <15 >15		Integrative analysis
Child’s identity^b^		
*He’s two and a half years old, but he’s like a baby... I have a LOT of work to do with him. If you look at it like that, everything is more difficult. EVERYTHING is more difficult (M8)* *For me, C16 is a normal child. The only thing C16 doesn’t do is walk. She feeds well, sleeps well. She communicates with you. She’s a normal child. She communicates, talks, calls you, plays with you, hugs you, kisses you (M16)*	*The only thing that makes it different is that she wears that device on her stomach, that’s all. She’s super smart. There was NOTHING on her little head when I did the MRI. So there are no other problems, no illnesses, which is why today I see it as normal (M10)* *In reality, she’s considered a normal child. Despite her sight, she doesn’t stop doing anything that a normal child does (M19)*	When the scores were higher, the participants showed a perception of normality in the child’s life and daily routine. To a lesser extent, limitations or vulnerabilities were also presented, but with the understanding that the child and their life are not restricted to the chronic condition. The participants with the lowest scores, although indicating positive aspects of the child and their daily life, emphasized limitations and weaknesses in their speeches, showing the impairment caused by the health condition.

Note: ^a^: Quantitative stage;

^b^: Qualitative stage.

There is therefore a co-occurrence of the caregivers’ view of their child. At the same time as they say that their children have similar behaviors and abilities to other children of the same age, using expressions such as *She’s a girl like any other (M10), there’s no difference (M11), she doesn’t stop doing anything that a normal child does (M19*), they make explicit the limitations that the chronic condition imposes on them. The speech fragments of M21 and M31 allow us to verify this. In the case of M21, the children he plays with accept him very well and the child himself *has not noticed this difference in him*. M31, although stating that C31 *is now a normal child*, explains what normal means to her, referring *to doing whatever she wants within her limits* and, therefore, *she is a normal child as far as possible*.

Even the families who had a low score (<15) on the CDL had a positive outlook on their children’s condition. They did this by again using the potential and abilities they could recognize in their daily experience with the children, which allowed them to build a life that was close to what is expected for children of the same age without alterations to their state of health. This is evidenced by M9 saying that her son C9 has a medical diagnosis of agenesis of the corpus callosum, *but he is very good at keeping things, he doesn’t forget! He seems to have an extra IQ*. For M16, C16’s cerebral palsy and the use of a technological device do not make it impossible for the child to carry out common activities for his age, *she communicates, talks, calls you, plays with you, hugs you, kisses you. The only thing C16 doesn’t do is walk*.

However, there are aspects that point to a view of the child that reinforces their vulnerabilities and limitations. The speeches express the children’s dependence on daily activities and maintaining life, demanding time and dedication for care actions. Due to the high degree of neurological impairment, the families emphasize that their children’s health is below what is expected for their age, and in this situation, care actions become more difficult. We can recognize this understanding when they say that the *child is two and a half years old, but he’s like a baby... I have a LOT of work to do with him. If you look at it like that, everything is more difficult* (M8). The child’s dependence on me to carry out daily activities can be seen in the following speeches: *If I don’t get to her and do it, she’ll stay there, she won’t talk. Every three hours there’s food, because I don’t know when she’s going to get hungry* (M22); *C35 is dependent on everything, so we end up living depending on him* (M35), which highlight the children’s less active behavior.

The View of the Impact of the Condition scale relates to the severity of the chronic condition in the family environment and the repercussions for the child and family members. Among the 53 families who took part in the study, three (5.67%) scored 30 on the Impact of the Condition scale, 18 (33.96%) scored less than 30 and 33 (60.37%) scored more than 30, indicating a greater impact on the family context. The average score was 27.18 and the scale mean was 30. There was a statistically significant correlation between the VDC and VIC scores (p < 0.001, Pearson’s coefficient –0.497).

Even though the scores were lower in relation to the VIC, there were discourses that expressed a more positive view of the impact of the condition (Chart 2). These families share the view that the chronic condition has had fewer repercussions for the family and the child, with lower levels of concern about their child’s condition at the time of data collection, especially when compared to the time of diagnosis. This can be seen in the fragment of M5’s speech when she says that *At FIRST, it wasn’t very easy, but over time we manage to fit everything in. The children’s progress was also seen by family members: nowadays she can stand up on her own, she even takes a few steps on her own (M5); and M12 when he said that Before he didn’t raise his arm, now he raises his arm*. Another aspect worth noting is that these children were using less complex technological devices.

**Chart 2:** Quantitative results obtained using the Impact of Condition View Scale and qualitative data from the View of Illness category - Belo Horizonte, MG, Brazil, 2020.

Score Scale View of the Impact of the Condition (minimum: 10 maximum: 50; mean: 30)^a^ <30 >30		Integrative analysis
View of the disease^b^		
*Thank God she’s improved a lot, nowadays she can stand up on her own, she even takes a few steps on her own (...) I’ve accompanied her when she was intubated, I’ve accompanied her when she was parenterally dependent... So there are no difficulties nowadays. The difficulty has passed.” (M5)* *Today he’s very well. Before he wouldn’t raise his arm, now he raises his arm (...) He’s totally different now. I already dress the left side, and he raises his right arm. He can’t lift it all the way, but he can. (M12)*	*Because before, we couldn’t accept that such a small child had such a serious illness. We’re afraid that over time it will get worse, but we can’t let it get to us. We’re worried about his medication and hope that later on we’ll be able to reverse something in his condition. (M11)*	Participants with lower scores indicating a lower level of concern about the child’s health condition also expressed less concern through their speeches. They say that this has improved over time as the child has developed and there has been less demand for care. Participants with higher scores emphasized the repercussions for the family of caring for a child with a chronic condition. Non-acceptance of the chronic condition also recurred in the speeches.

**Note:** ^a^: Quantitative stage;

^b^: qualitative stage.

On the other hand, some families perceive their child’s chronic condition as very serious and with the potential to compromise their lives, describing how difficult it is to live with their child’s situation, which makes it impossible for the family to have a life as close to normal as possible. M08’s speech reveals the difficulty in accepting the child’s condition: *It’s not easy, it’s difficult, I didn’t want it... I want it to be reversed, I’m looking for a cure, I don’t accept it*, making it clear that she is uncomfortable with the situation she is experiencing. In addition, guilt for the child’s health condition was also verbalized, as seen in M45’s statement that: *I realized that it was my fault, I didn’t want to accept it, and M46: Wow, my world fell apart, because when he was born and went to the ICU I was already blaming myself, to this day I have this feeling that it’s my fault that he turned out like this* (M46).

The Management Effort scale refers to the time used and the effort required to provide care at home. Of the 53 family members who answered this scale, five (9.43%) had a score of 12, nine (16.98%) had a score of less than 12 and thirty-nine (73.59%) had a score of more than 12, with 12 being the average for the scale. The average score obtained was 14.16, meaning that, on average, the family needs to provide more attention and time to carry out care at home in order to meet the care needs of the child with a chronic condition. Regardless of the score on the Management Effort scale, the participants stressed in their speeches that the intensity of the actions carried out to meet the children’s needs brings overload, exhaustion, demands effort and time to be able to meet the care demands (Chart 3).

**Chart 3:** Quantitative results obtained using the Management Effort Scale and qualitative data from the Management Mentality category – Belo Horizonte, MG, Brazil, 2020.

Management Effort score (minimum: 4 maximum: 20; mean: 12)^a^ <12 >12		Integrative analysis
Management mentality^b^		
*I’m going to say it’s tiring, because it is tiring. Because there are appointments, there are tests to do. I have to write everything down so I don’t forget, I have to carry it with me. It’s all me, so it’s even more tiring (M33)*	*I don’t stop at home, I go out with C35 every day to take him to his therapies. It’s tiring for him too and there’s the other one too, who goes along. I have to take the youngest to his brother’s therapy too (M35)*	Participants with lower and higher scores on the management scale emphasize the intense routine involved in caring for the children. These speeches highlight the effort and time spent by the family to meet the demands of care, similar to what was identified in the interviews with participants who had higher scores.

**Note:** ^a^: Quantitative stage;

^b^: qualitative stage.

The centrality of care for the main caregiver is evident. In this study, in 47 families the mother occupied this role. They describe the strategies used to ensure that all the care actions take place, such as: *writing everything down so I don’t forget* (M33), I have to set up my timetable according to C35’s therapies (M35). And they recognize that the effort put into managing him tires them out even more (M33), *it’s very hard, it’s hard for me, I think it’s hard for him too* (M35). However, despite the effort involved in caring, the results also show that it is for their children and in their interest that parents act and, in this way try to deal with the difficulty of their caring role.

## DISCUSSION

The data presented in this study allows us to delve deeper into the family context as well as into aspects arising from family members’ perspectives on children with chronic conditions and how they live with these children. They also explain the need for changes resulting from this care. The articulation of the quantitative and qualitative findings reinforces the dynamic nature of what families experience and the contribution that the mixed approach has made, allowing for the identification of aspects within the scope of their subjectivities, but which may require attention due to their potential to produce more significant changes in the family’s perspective on the situation.

The participants’ speeches converge with the results of the measures used, but they do not fail to make explicit the events they experience which, although they do not seem to be decisive in changing the result obtained with the measures, allow us to recognize that even a positive perspective of the situation of caring for a child with a chronic condition comes with tensions and daily difficulties, sometimes in the realm of subjectivity.

Guilt is mentioned and, as a negative feeling of family members, it is very relevant in nursing and is still very much hidden. Nursing intervention to alleviate this feeling is one of the biggest challenges for nurses in general, and particularly for parents of children with chronic conditions^(19)^. In this context, it is essential for nursing professionals to help reduce the negative feelings expressed by family members^(20)^. This can be achieved by providing clear guidance on the diagnosis and the care needed at home, as well as establishing a welcoming relationship by listening attentively to the family’s experiences^(20)^. In this way, nursing professionals can develop strategies that promote care not only for the child, but also for the whole family in the home environment^(21)^.

The perspective of normalization identified through the scores on the Child’s Daily Life scale and in the participants’ speeches is a finding that corroborates the results of other investigations carried out with CCC families^(22,23)^. In this study, the participants explain their view of normalizing their child and their family’s daily life. At the same time, they express their understanding that their children have a chronic health condition and that their lives and their children’s lives are not limited to the child and the care required. These findings allow us to infer that the identification of a family perspective of normalization in relation to the child can be a family resource for continuing with the care and also be a clue that the family is moving towards effective management.

In this study, the participants explain their vision of normalization in relation to the child and their daily family life. At the same time, they express their understanding that their children have a chronic health condition and that their lives and their children’s lives are not limited to the child and the care required. These findings allow us to infer that the identification of a family perspective of normalization in relation to the child can be a family resource for continuing with the care and also be a clue that the family is moving towards effective management.

Lower scores on the Child’s Daily Life scale were identified in participants who emphasized during the interviews the child’s weaknesses and the impairment caused to their health by the chronic condition. Although they also identified positive aspects, this was not the aspect that emerged most strongly from their speeches, in line with the quantitative findings.

The findings show that 33 participants had higher scores on the View of the Impact of the Condition scale, demonstrating a difficulty in accepting the child’s health condition. It should be noted that the difficulty of acceptance and the scores indicating a greater impact of the chronic condition on the family’s life were identified regardless of the complexity of the health condition and/or the intensity of the care required. It can be said that, even after some time has passed, the chronic condition still needs to be accepted by the participants and, as part of their view of the impact of the condition, it can be a hindrance to family management.

There were situations that make some families vulnerable to be highlighted, such as the lack of acceptance of the health condition, the identification of the child as fragile and with weaknesses and the overload caused by caring. Even though the family is recognized as a system with the potential to adapt to different situations, the maintenance of vulnerabilities over time means that families’ existing potential is exhausted and compromises their ability to care^(24)^.

A cross-cultural research study in 11 countries found that, in general, countries had positive family management, while countries such as Ireland, Italy, Japan and Korea had more problematic family management, in which the presence of a child with Down’s Syndrome had a negative impact on the family context^(25)^. The qualitative data contributes to understanding the situations experienced by the families in terms of the efforts made to manage the family. Reports of the intense care routine in the home environment and in specialized health care were evident in the participants’ speeches, regardless of the score on the Management Effort scale.

It is remarkable that participants with scores that indicated a greater effort to manage emphasized the intensity of the care routine and what they had to do to maintain it. These situations were more frequent in the speeches of participants whose children had significant impairment of their motor and cognitive functions without improvements that reduced the need for care until the time of the study. These findings are similar to those found in a study with family members of children with Juvenile Idiopathic Arthritis. The mothers highlight how difficult and challenging it is to provide the care necessary to maintain their children’s lives, and also describe the need to prioritize caring for their child over other domestic activities. They explain that the time spent protecting and promoting their children’s well-being leads to personal and professional sacrifices^(26)^.

It should be noted that each family has unique characteristics and deals with chronic conditions according to specific social, individual and/or cultural patterns and behaviors. In this context, nursing plays a crucial role in providing a deeper understanding of the needs of affected children and their families^(27)^.

Recognizing that CCCs often face significant challenges in their lives, having a supportive family can help them cope better with these challenges, improving their self-esteem and confidence, creating a more positive and stable environment at home. Therefore, investing in support for CCC families is essential to improve the quality of life of these children and help them achieve a greater degree of independence and self-management. Therefore, the family’s ability to manage the care situation has a positive influence on quality of life and strengthens the independence and self-management skills of children and adolescents with chronic conditions^(28)^.

The limitation of this study refers to the fact that the participating children have different chronic conditions, with different severities. Although the care demands may be similar, they may differ in terms of the frequency and complexity of the actions required.

## CONCLUSION

As a conclusion, families have a positive view of the situation of caring for CCC who have left the NICU at home. The view of normality accepted by the families during the course of continuous and prolonged care allows the family environment in which the child is inserted to become more welcoming and this has a positive impact on the child’s living conditions, feeling supported by everyone involved in the care. However, the speeches show that some families experience daily battles to care for their children, either due to the child’s own clinical condition which is getting worse or due to the child’s growth, making daily care actions difficult.

By using the mixed method, this study produced more consistent findings and also made it possible to explain the repercussions of caring for children on family relationships. This understanding can guide professionals towards more appropriate family interventions, since nurses can incorporate the perspective of the main caregivers into the planning and execution of care, as well as offering emotional and practical support. It is recommended that professionals orient their practices towards helping family members recognize their children’s potential, without disregarding the impact of the condition on the family and the need for them to be supported so that they can ensure care at home.
